# Supplementary material for: Dehydroisohispanolone as a Promising NLRP3 Inhibitor Agent: Bioevaluation and Molecular Docking
Source: Pharmaceuticals (Basel). 2022 Jul 2;15(7):825. doi: 10.3390/ph15070825 (PMC9316970; doi:10.3390/ph15070825)
Supplement: Supplementary file 1 [file pharmaceuticals-15-00825-s001.zip › pharmaceuticals-1767852-supplementary.pdf]

Dehydroisohispanolone as a promising NLRP3 inhibitor agent. Bioevaluation and Molecular Docking.

Laura González-Cofrade[1], Irene Cuadrado[1], Angel Amesty[2], Ana Estévez-Braun[2,*], Beatriz de las Heras[1, *], Sonsoles Hortelano[3,*]

[1]Departamento de Farmacología, Farmacognosia y Botánica, Facultad de Farmacia, Universidad Complutense de Madrid (UCM), Plaza Ramón y Cajal s/n-28040, Madrid, Spain; lagonz11@ucm.es (L.G-C); icberrocal@ucm.es (I.C.); lasheras@ucm.es (B. de las H.).

[2]Departamento de Química Orgánica. Instituto Universitario de Bio-Orgánica Antonio González, Universidad de La Laguna, Avda. Astrofísico Francisco Sánchez Nº 2, 38206, La Laguna, Tenerife, Spain; aarnesty@ull.es (A.A.); aestebra@ull.es (A. E-B.)

[3]Unidad de Terapias Farmacológicas, Área de Genética Humana, Instituto de Investigación de Enfermedades Raras (IIER), Instituto de Salud Carlos III, Carretera de Majadahonda-Pozuelo Km 2-28220, Madrid, Spain; shortelano@isciii.es (S.H.)

Content

Preparation of deshydroisohispanolone (DIH)

^{1}H NMR ($CDCl_3$) of deshydroisohispanolone — Figure S1

^{13}C NMR ($CDCl_3$) of deshydroisohispanolone — Figure S2

NLRP3 inflammasome inhibition by MCC950 — Figure S3

Raw data of Western blots — Figure S4-S10

Primers used and their sequences — Table S1

Antibodies used in Western Blot analysis — Table S2

Preparation of deshydroisohispanolone (DIH).

Deshydroisohispanolone was obtained from the natural diterpene hispanolone following the procedure described in reference [21]. Thus, to 3.0 g (9.45 mol) of hispanolone in 175 mL of EtOH were added 10 mL of concentrated HCl and the reaction mixture was heated under reflux for 18 h. Next, this was treated with 100 mL of H_2O and extracted with CH_2Cl_2 (3 x 30 mL). The organic phases were collected, dried over anhydrous $MgSO_4$, filtered, and the solvent removed. The residue was purified by column chromatography with hexanes-EtOAc (95:5) to yield 0.14 g (1.0 %) of deshydroisohispanolone (DIH) and 1.3 g of deshydrohispanolone (46%) as yellow oils.

DIH: $[\alpha]^{20}{}_D$ -91 (*c* 0.9, $CHCl_3$); IR (neat) ν_{max} 3150, 2970, 2850, 1665, 1505, 1465, 1305, 1160, 1030, 995, 765 cm^{-1}; ^1H NMR ($CDCl_3$, 500 MHz) δ 7.26 (1H, bs, H-15), 7.11 (1H, bs, H-16), 6.18 (1H, s, H-14), 3.53 (1H, d, *J* = 14.8 Hz, H-6a), 3.25 (1H, d, *J* = 14.8 Hz, H-6b), 2.33 (2H, m, H-12), 1.86 (1H, dd, *J* = 13.7, 1.4 Hz, H-5), 1.59 (3H, m, H-2, H-1), 1.43 (1H, dd, *J* = 13.2, 1.4 Hz, H-3), 1.32 (1H, td, *J* = 13.4, 5.1 Hz, H-8), 1.19 (1H, td, *J* = 13.0, 5.1 Hz, H-8), 1.07 (3H, t, *J* = 7.7 Hz, H_3-17), 0.99 (3H, s, H_3-20), 0.98 (3H, s, H_3-19), 0.91 (3H, s, H_3-18); ^{13}C NMR ($CDCl_3$, 100 MHz) δ 204.7 (C, C-9), 158.6 (C, C-11), 142.5 (CH, C-15), 139.1 (CH, C-16), 130.8 (C, C-7), 124.0 (C, C-13), 111.2 (CH, C-14), 48.4 (CH, C-5), 44.2 (C, C-10), 41.7 (CH_2, C-3), 33.7 (CH_2, C-1), 33.7 (C, C-4), 32.3 (CH_3, C-19), 28.1 (CH_2, C-6), 27.8 (CH_2, C-12), 22.2 (CH_3, C-20), 20.8 (CH_2, C-8), 18.3 (CH_2, C-2), 17.2 (CH_3, C-18), 12.4 (CH_3, C-17); EIMS *m/z* 300 ($[M^+]$, 100) 285 (8), 271 (11), 267 (10), 229 (15), 149 (32), 147 (30), 81 (54); HRESIMS *m/z* 323.1981 $[M+Na]^+$ (calcd for $C_{20}H_{28}O_2Na$, 323.1987).

Figure S1: ¹H NMR ($CDCl_3$, 500 MHz) of deshydroisohispanolone.

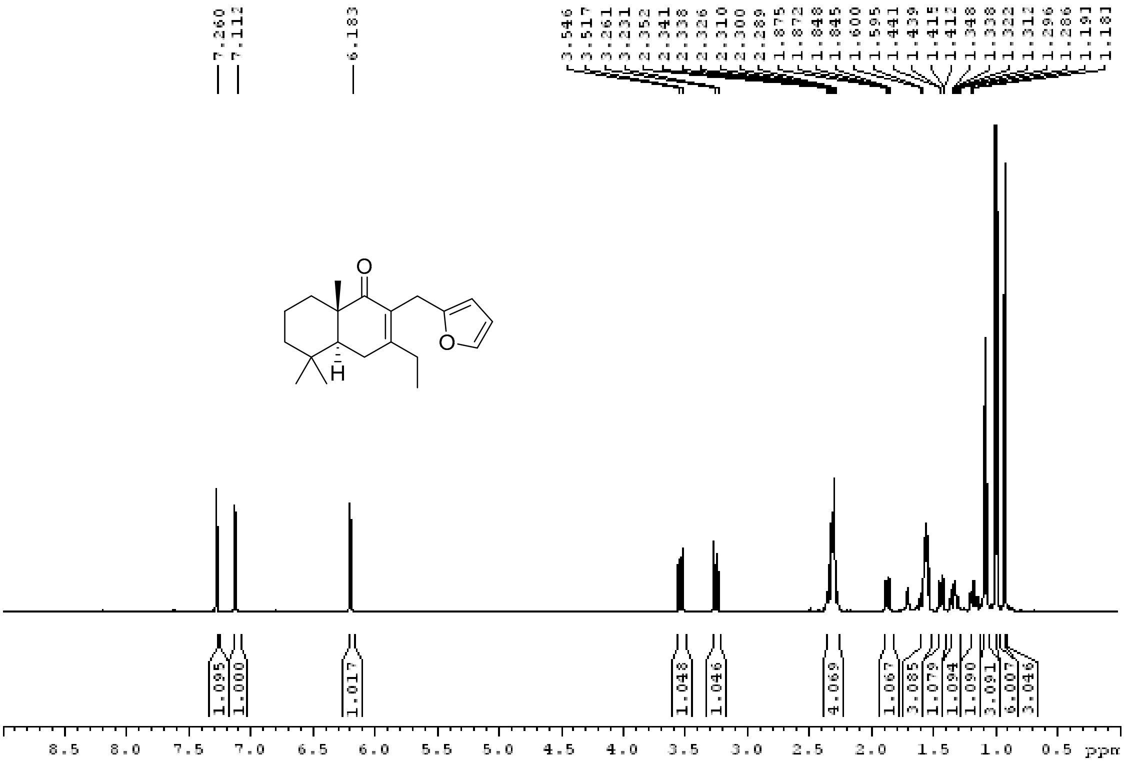

Figure S2: ¹³C NMR ($CDCl_3$, 100 MHz) of deshydroisohispanolone.

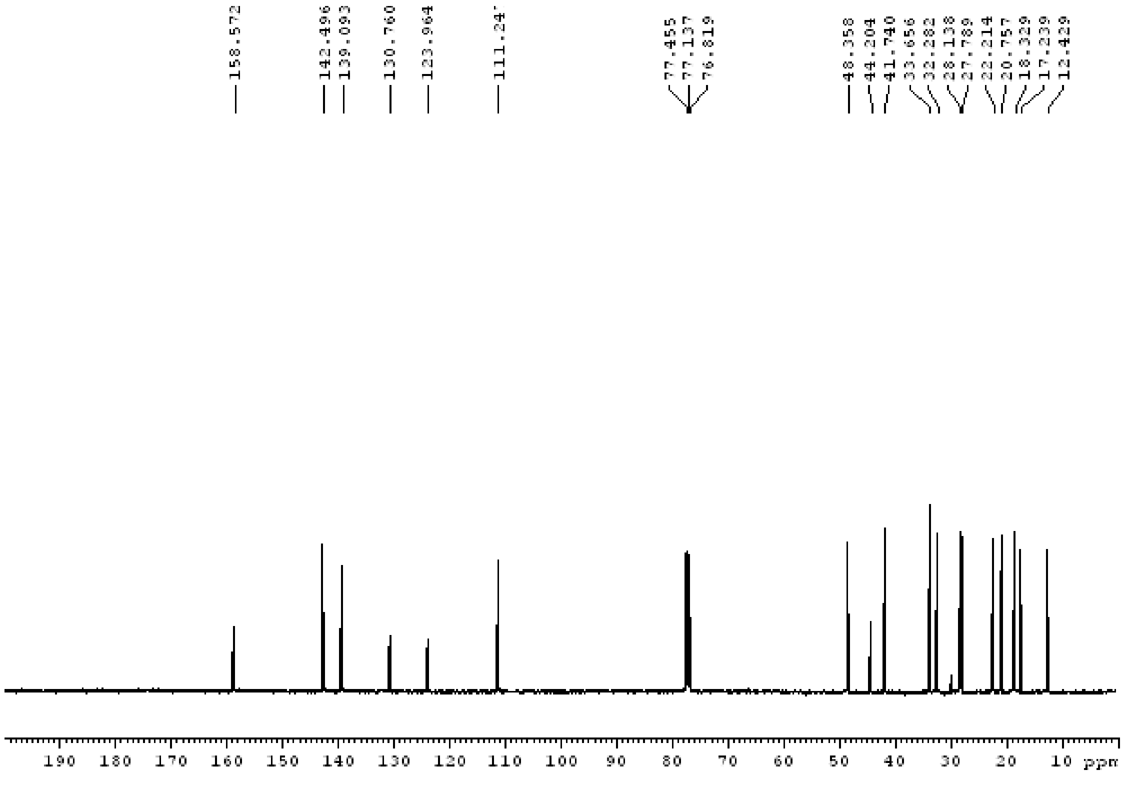

Figure S3: NLRP3 inflammasome inhibition by MCC950. MCC950 was used as a positive control for NLRP3 inflammasome inhibition. (A, B, C) LPS-primed J774A.1 macrophages were stimulated with Nig in presence of DIH (10, 20 μM) or MCC950 (10 μM) and IL-1β release (A), Caspase-1 activity (B) and LDH release (C) were then measured as described. (D) LPS-primed BMDMs were stimulated with Nig in presence of DIH (10, 20 μM) or MCC950 (10 μM) and IL-1β release was measured. Results are expressed as means ± SD (n = 3). *p < 0.05 and ***p < 0.001 vs. LPS + Nig treatment.

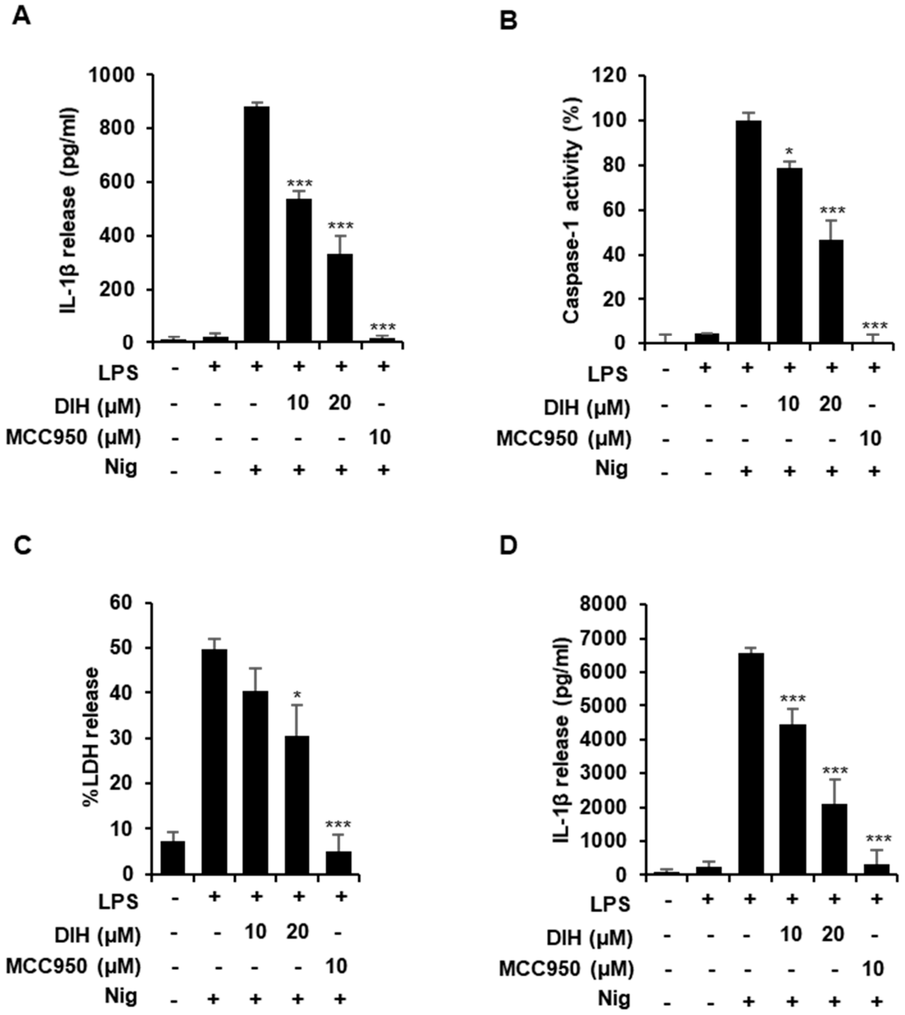

Figure S4. Original western blots for two repeats of Figure 2D. Whole blot of cell lysates after cutting membrane at molecular weight 35 kDa and 28 kDa for β-actin (42 kDa) and pro-IL-1β (31 kDa); and supernatants after cutting membrane at 28 kDa for cleaved IL-1β (17 kDa).

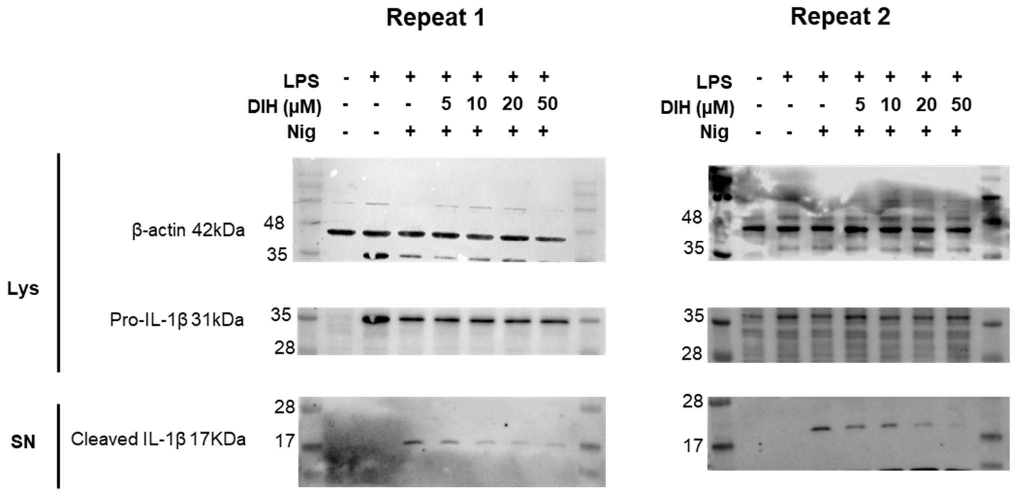

Figure S5. Original western blots for two repeats of Figure 2E. Whole blot of cell lysates after cutting membrane at molecular weight 35 kDa and 28 kDa for β-actin (42 kDa) and pro-IL-1β (31 kDa); and supernatants after cutting membrane at 28 kDa for cleaved IL-1β (17 kDa).

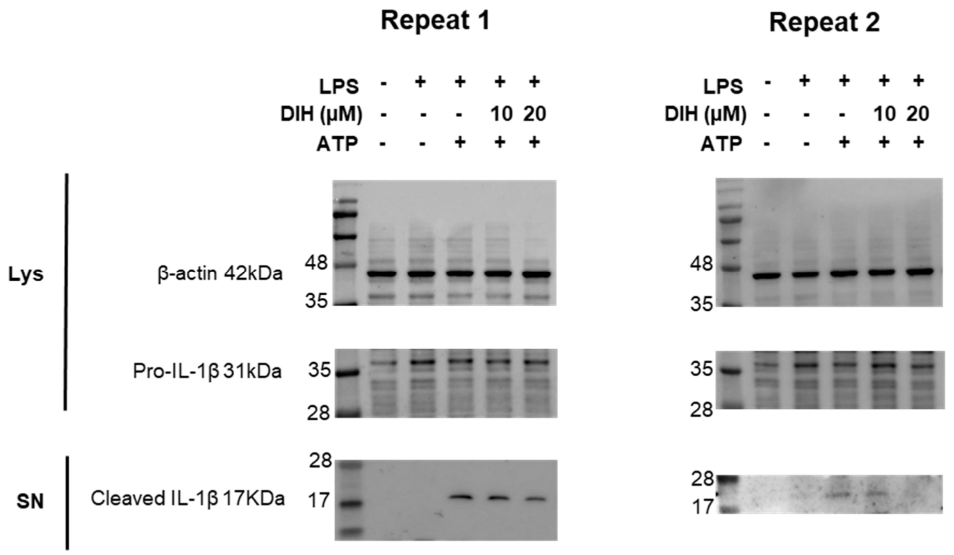

Figure S6. Original western blots for two repeats of Figure 2F. Whole blot of cell lysates after cutting membrane at molecular weight 35 kDa and 28 kDa for β-actin (42 kDa) and pro-IL-1β (31kDa); and supernatants after cutting membrane at 28 kDa for cleaved IL-1β (17 kDa).

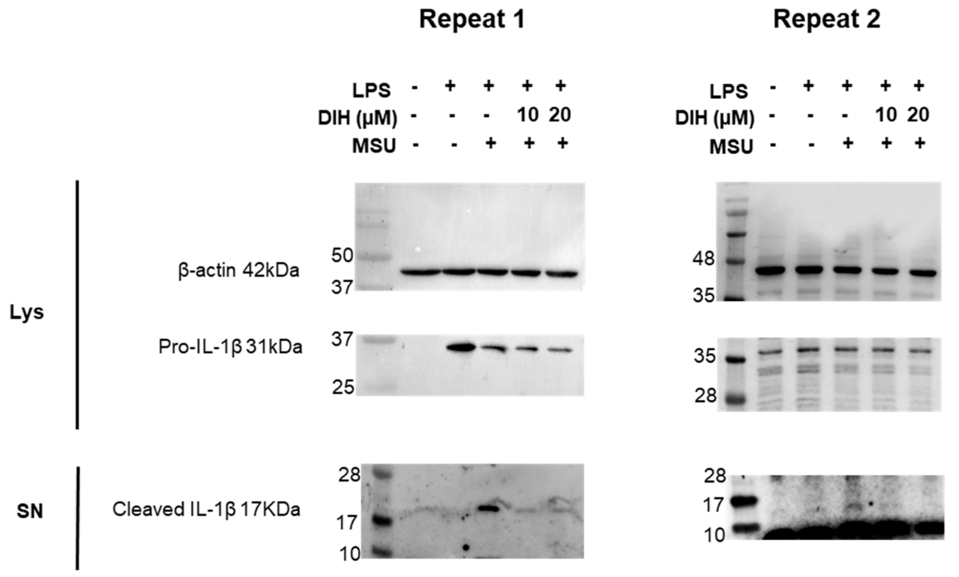

Figure S7. Original western blots for two repeats of Figure 3B. Whole blot of cell lysates after cutting membrane at molecular weight 48 kDa for pro-caspase-1 (45 kDa) and β-actin (42 kDa); and supernatants after cutting membrane at 10 kDa for caspase-1 p10 (10 kDa).

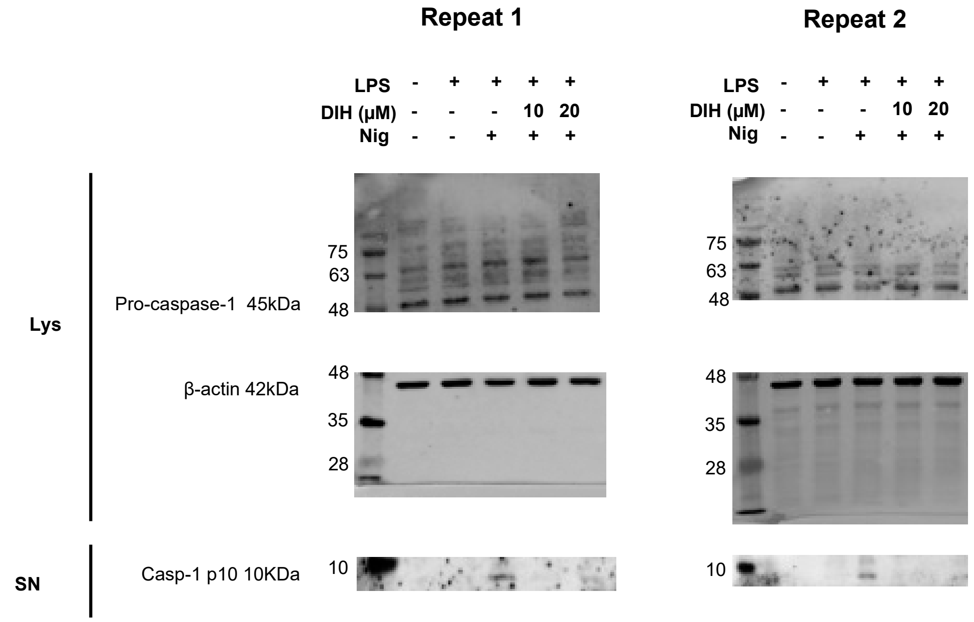

Figura S8. Original western blots for two repeats of Figure 4B. Whole blot of cell lysates after cutting membrane at molecular weight 48 kDa and 35 kDa for GSDMD (53 kDa), β-actin (42 kDa) and GSDMD-N (32 kDa).

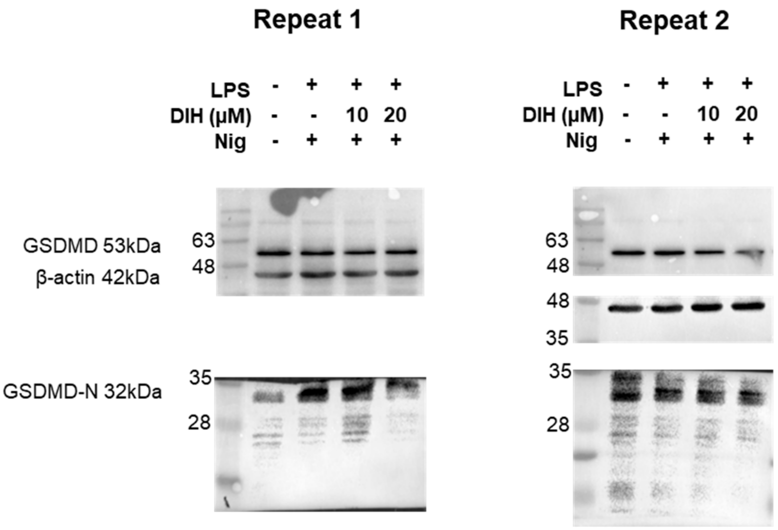

Figure S9. Original western blots for two repeats of Figure 5B. Whole blot of cell lysates after cutting membrane at molecular weight 63 kDa, 35 and 28 kDa for NLRP3 (103 kDa), β-actin (42 kDa) and ASC (24 kDa).

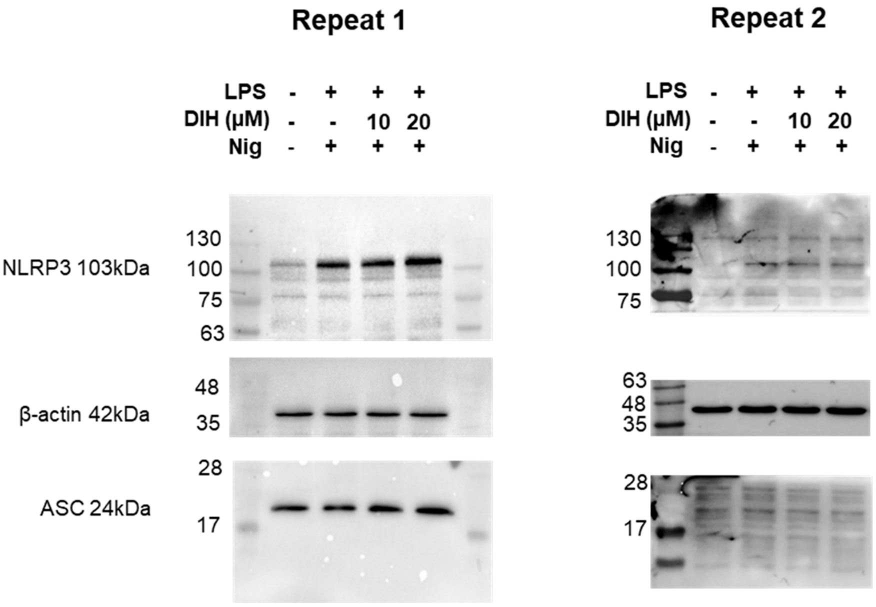

Figure S10. Original western blots for two repeats of Fig. 6B. Whole blot of cell lysates after cutting membrane at molecular weight 63 kDa, 48, 35 and 28 kDa for NLRP3 (103 kDa), pro-caspase-1 (45 kDa), β-actin (42 kDa), pro-IL-1β (31kDa) and ASC (24 kDa).

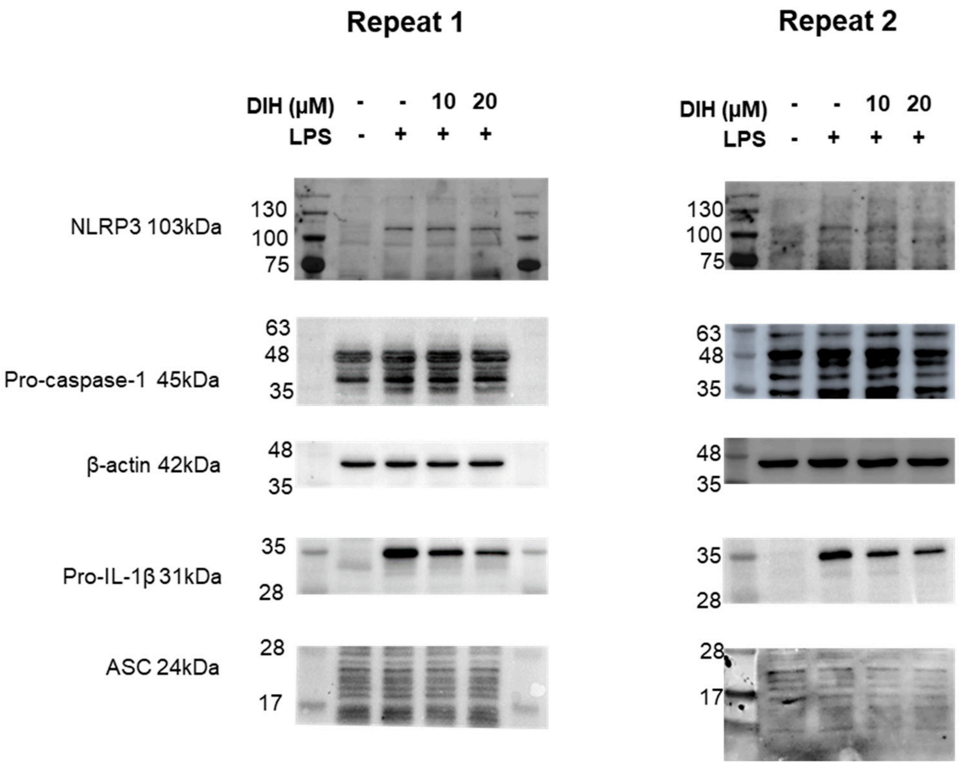

Table S1. Primers used and their sequences.

Gene	Forward primer 5'-3'	Reverse primer 5'-3'
**NOS-2**	TCCACAGTATGTGAGGATCAAAAAC	ATGTGGCCTTGTGGTGAAGAGT
**COX-2**	GCTGTACAAGCAGTGGCAAAG	GCGTTTGCGGTACTCATTGAGA
**TNFα**	CATCTTCTCAAAATTCGAGTGACAA	TGGGAGTAGACAAGGTACAACCC
**IL-6**	GAGGATACCACTCCCAACAGACC	AAGTGCATCATCGTTGTTCATACA
**Pro-IL-1β**	TCTTTGAAGTTGACGGACCC	TGAGTGATACTGCCTGCC TG
**NLRP3**	AGCCTTCCAGGATCCTCTTC	CTTGGGCAGCAGTTTCTTTC
**IL-18**	TGGTTCCATGCTTTCTGGACTCCT	TTCCTGGGCCAAGAGGAAGTGATT
**Pro-caspase-1**	AGATGGCACATTTCCAGGAC	GATCCTCCA GCAGCAACTTC
**36B4**	AGATGCAGCAGATCCGCAT	GTTCTTGCCCATCAGCACC

Table S2. Antibodies used for Western Blot analysis.

Antibody	Source	Identifiers	Dilution
Anti-mouse IL-1β (goat polyclonal)	R&D systems	AF-401-NA	1:500
Anti-pro Caspase-1 + p10 + p12 (rabbit monoclonal)	Abcam	ab179515	1:1000
Anti-NLRP3/NALP3 (Cryo-2) (mouse monoclonal)	AdipoGen	AG-20B-0014	1:1000
Anti-mouse ASC/TMS1 (D2W8U) (rabbit monoclonal)	Cell Signaling Technology	67824T	1:1000
Anti-β-actin (mouse monoclonal)	Sigma	A5541	1:5000
Anti-GSDMD (rabbit monoclonal)	Abcam	ab209845	1:1000
Goat Anti-Rabbit IgG H&L (HRP)	Abcam	ab205718	1:10000
Anti-goat IgG HRP	R&D systems	HAF017	1:1000
Anti-mouse IgGκ BP-HRP	Santa Cruz	sc-516102	1:5000
